# Cerebral and Peripheral Immune Cell Changes following Rodent Juvenile Traumatic Brain Injury

**DOI:** 10.3390/brainsci14040398

**Published:** 2024-04-19

**Authors:** Allie M. Smith, Erin B. Taylor, Ruth J. Brooks, Christiano Dos Santos e Santos, Bernadette E. Grayson

**Affiliations:** 1 Department of Neurology, University of Mississippi Medical Center, Jackson, MS 39216, USA; asmith40@umc.edu (A.M.S.); rbrooks1@umc.edu (R.J.B.); cdsantos@mdanderson.org (C.D.S.e.S.); 2 Department Physiology and Biophysics Behavior, University of Mississippi Medical Center, Jackson, MS 39216, USA; ertaylor@umc.edu; 3 Department of Anesthesiology, University of Mississippi Medical Center, Jackson, MS 39216, USA

**Keywords:** immune cells, inflammation, pediatric TBI

## Abstract

Traumatic brain injury (TBI) is one of the leading causes of death and disability. TBI is associated with neuroinflammation, but temporal changes in immune and inflammatory signaling following TBI have not been fully elucidated. Furthermore, there have been no previous studies on changes in immune cell populations following TBI via the Closed Head Injury Model of Engineered Rotational Acceleration (CHIMERA). The current study aimed to determine the time course changes to inflammatory marker mRNA expression in the acute period following TBI in juvenile rats and to determine acute changes to brain and circulating immune cell populations. For this study, post-natal day (PND)-30 male Long Evans rats sustained a TBI or Sham TBI and were euthanized at 0, 3, 6, 12, 24, or 96 h post-injury. Prefrontal cortex and hippocampus samples were used to determine mRNA expression changes of inflammatory factors. The mRNA expression of the pro-inflammatory cytokine TNF-α was significantly elevated at 6 h post-injury in both regions evaluated. To evaluate immune cell populations, male Long Evans rats were euthanized at 48 h post-injury, and brain and blood samples were used for cell sorting by marker-specific antibodies. In the peripheral blood, there was an elevation in CD3^+^ total T cells, CD45R^+^ total B cells, and CD3^+^CD4^+^ helper T cells in the TBI subjects. However, there were no changes to natural killer cells or CD3^+^CD8^+^ cytotoxic T cell populations. In the brain, there was a reduction in CD11b/c^+^ monocytes/macrophages, but no changes in other immune cell populations. At 48 h post-injury, the TBI subjects also demonstrated expansion of the thymic medulla. These changes in the cerebral and blood immune cell populations and thymic medulla expansion may implicate the subacute recovery timeframe as a vulnerable window for the immune system in the pediatric population.

## 1. Introduction

Traumatic brain injury (TBI) is a significant public health concern that can have debilitating consequences, particularly in the juvenile population, in which TBI is one of the leading causes of death and disability [1]. An insult to the brain initiates both primary and secondary injury cascades. The primary injury consists of mechanical damage to the brain due to impact with the skull protuberances [2,3,4]. The secondary injury cascade, which occurs in the minutes and hours following the injury, can consist of blood-brain barrier (BBB) disruption, inflammatory process, and cell death [5]. Neuroinflammation can produce both beneficial and detrimental changes to neural tissue following injury, and due to this duality, inflammatory processes have often been viewed as a target for a therapeutic option for TBI [5,6]. However, the functions and processes of neuroinflammation following brain injury are not entirely understood.

Neurological manifestations of concussion focus attention on the neural and vascular components of the brain; however, parenchymal damage invites a cascade of immune cell participation in the response and debris clearance following TBI. Using adult models, some laboratories have similarly focused on parsing the immune populations that are affected by TBI [7,8,9,10]. Unsurprisingly, shifts in immune cell populations in peripheral blood after TBI have been identified acutely, with some enduring results chronically [11,12,13,14]. Groups that have used the CHIMERA to produce mild injury have not previously reported any studies evaluating central and peripheral immune cell population shifts. Furthermore, limited studies exist in a juvenile TBI model evaluating immune cell infiltration within the cerebral cortex or circulating immune cell populations [14,15]. The gap in knowledge regarding central and peripheral immune cell changes in the juvenile population is critical to fill because the inhibitory (GABAergic) inputs to the prefrontal cortex in the juvenile are not solidified at this developmental time, suggesting that damage to the cytoarchitecture of the brain and/or immune cell infiltration could significantly modulate the neural networks. Furthermore, as opposed to an adult, where thymic T cell processing has peaked and thymic involution is actively occurring, the thymus continues to educate and mature T cells in a juvenile. As a result, this developmental stage (the juvenile age) is particularly vulnerable.

The current study investigates acute cerebral and peripheral immune cell changes using flow cytometry. We report time course changes in relevant immune-related genes. We also analyze histologic sections of the thymus and spleen. Taken together, significant changes to immune cells, both centrally and peripherally, and relevant lymphoid organs occur 48 h after injury.

## 2. Methods

### 2.1. Institutional Animal Governance

Procedures for animal use were approved by the University of Mississippi Medical Center (UMMC) Institutional Animal Care and Use Committee, Protocol #1589. These animal-use procedures complied with the National Research Council’s Guidelines for the Care and Use of Laboratory Animals.

### 2.2. Animals

Post-natal day (PND)-20 male Long Evans rats were obtained from commercial sources (RRID: RGD_5508398, Inotiv, Indianapolis, (IN), USA). The animals were group-housed in the UMMC vivarium and were maintained on a 12 h/12 h light/dark cycle with 50–60% humidity at 25 °C. The animals were given ad libitum access to a standard chow diet (#8640, Inotiv, 3.0 kcal/g; 17% fat, 54% carbohydrate, 29% protein) and were randomly assigned to either Sham or TBI groups.

### 2.3. CHIMERA Procedures

On PND 30, rats were placed in an induction chamber and anesthetized using 3.5% isoflurane in 100% oxygen. Both Sham and TBI subjects received subcutaneous injections of carprofen (5 mg/mL) for pain management following the procedures. After animals were fully anesthetized and nonresponsive to a toe pinch, they were placed on the CHIMERA animal holder at a 45° angle in the supine position and were secured to the animal holder using Velcro straps; while the animals were on the CHIMERA apparatus, anesthesia was maintained via nosecone. The TBI subjects’ heads were aligned so that the piston would deliver the injury between the eyes and the ears in the center of the head. Immediately prior to the piston deployment, the nosecone was removed briefly, but the subjects were still fully anesthetized. Sham animals were treated the same in all respects, but they were placed beside the animal holder so that they did not sustain a TBI. Immediately following TBI or Sham procedures, the animals were placed in a recovery chamber in the supine position, and time to righting and walking were analyzed following placement in the recovery chamber while the animal was awakening from anesthesia. Time to righting is a measure of consciousness, and it is the latency to return to the prone position from the supine position after awakening from anesthesia. Time to walking is considered the use of all four limbs in an attempt to walk.

### 2.4. Study 1

The subjects (Total *n* = 21, *n* = 3 per group) sustained either a Sham TBI or a 0.6 J vertical TBI. TBI subjects were then euthanized immediately following TBI or 3 h, 6 h, 12 h, 24 h, or 96 h post-injury via conscious decapitation. Sham subjects were euthanized at 3 h post-Sham injury. Brains and terminal trunk blood were collected. Brains were flash-frozen in methylbutane, cooled on dry ice, and the prefrontal cortex and hippocampus were microdissected for RNA extraction.

### 2.5. Study 2

The subjects (Total *n* = 20; Sham groups: *n* = 10, TBI groups: *n* = 10) received either a Sham TBI or a 0.6 J vertical TBI on PND 30. On PND 32, animals were euthanized 3 h after the onset of the light cycle, brains were excised, and blood was collected for immune cell extraction and sorting. Spleens and thymuses were collected and used for H&E staining.

### 2.6. RNA Processing and Real-Time PCR

Brain tissue was flash-frozen on methylbutane and cooled on dry ice. The tissue was then stored in −80 °C until further processing. Using a QIAGEN miniprep RNA kit (QIAGEN, Inc., Valencia, CA, USA), RNA was extracted. Complementary DNA was transcribed using an iScript complementary DNA synthesis kit (Bio-Rad Laboratories, Hercules, CA, USA). Quantitative polymerase chain reaction was performed using TaqMan inventoried gene expression assays (Life Technologies, Foster City, CA, USA) on a Step-One Plus Real-Time PCR machine with StepOne Software (v2.3) (Applied Biosystems, Foster City, CA, USA)).

### 2.7. Cell Isolation Protocols

#### 2.7.1. Brain

Tissue was cut into approximately 1 mm × 1 mm pieces, and 5 mL of digestion solution (RPMI medium containing 10% fetal bovine serum (FBS), 10 μg/mL DNase, and 0.1% collagenase) was added. Samples were incubated at 37 °C for 30 min, mixed with a pipette, and incubated for an additional 30 min. Following incubation, digestive was filtered through a 70 μm filter and the filter was washed with 10 mL of 1× phosphate buffered saline (PBS), 2% FBS (wash buffer). The cell suspension was subsequently centrifuged at 300× *g* for 10 min. After discarding the supernatant, the cells were resuspended in 3 mL of 1× PharmLyse (BD Biosciences, Franklin Lanes, NJ, USA) to lyse erythrocytes for 5 min at RT. After incubation, 10 mL of wash buffer was added and the tubes were centrifuged at 300× *g* for 10 min. The pellet was then resuspended in 8 mL of 30% Percoll in RPMI. Five milliliters of Hank’s balanced salt solution (HBSS) was gently layered on top and the samples were centrifuged for 30 min at 800 rpm at 4 °C with no brake. The myelin middle layer was gently removed, and cells were resuspended in 30 mL HBSS. Samples were centrifuged for 50 min at 800 rpm at 4 °C. All but 5 mL of solution were removed, and cells were resuspended in 10 mL of wash buffer. Samples were centrifuged again, and cells were resuspended in wash buffer after the supernatant was discarded. Cells were centrifuged for 5 min at 350× *g* at 4 °C. The resulting cells were used in flow cytometric analyses.

#### 2.7.2. Peripheral Blood Leukocytes (PBL)

Erythrocytes were lysed using 1× PharmLyse (BD Biosciences Franklin Lanes, NJ, USA) according to the manufacturer’s instructions. Cells were washed and resuspended in PBS pH 7.4, containing 2% FBS and 0.09% sodium azide (stain buffer). An amount of 5 × 10^5^ cells were stained for 30 min on ice with immune cell-specific antibodies (BD Biosciences, Franklin Lakes, NJ, USA) as follows: CD8a (#561611), CD4 (#554837), CD3 (#554833), CD11b/c (#743980), CD161a (#555009), or CD45RA (#554881) at a concentration of 1:100 diluted in stain buffer. Cells were washed two times with 2 mL stain buffer and centrifuged at 350× *g* for 5 min at 4 °C. Cells were resuspended in 400 μL of stain buffer and immediately analyzed using a BD FACSymphony A3 Flow Cytometer (BD Biosciences, Franklin Lanes, NJ, USA) at the UMMC Flow Cytometry and Cell Sorting Core Facility. Data were analyzed using FlowJo software version 10.8.2. Gating strategy for both the brain and PBL are included in Supplemental Figure S1.

### 2.8. Paraffin Embedding and Standard Stains

Paraformaldehyde post-fixed spleens were subjected to standard paraffin-embedding and then sectioned at 5 µm onto glass slides for staining with hematoxylin and eosin (H&E). Bright-field and fluorescent microscopy photographs were obtained with 10× magnification.

### 2.9. Cytokine Analyses

V-PLEX custom rat biomarker assay was used to measure pro-inflammatory cytokine IL6 (#K153A0H-1, Mesoscale Discovery Systems, Rockville, MD, USA). Samples were diluted according to the manufacturer’s standard protocol.

### 2.10. Statistical Analyses

Statistical analyses were performed using GraphPad Prism version 10.0.2. One-way ANOVA with repeated measures was used to determine differences of time. Multiple comparisons were made to the Sham group. Unpaired Student’s *t* test was used to determine differences of injury. Means were considered significant at *p* < 0.05. The results are given as means ± SEM.

## 3. Results

**Figure 1:**
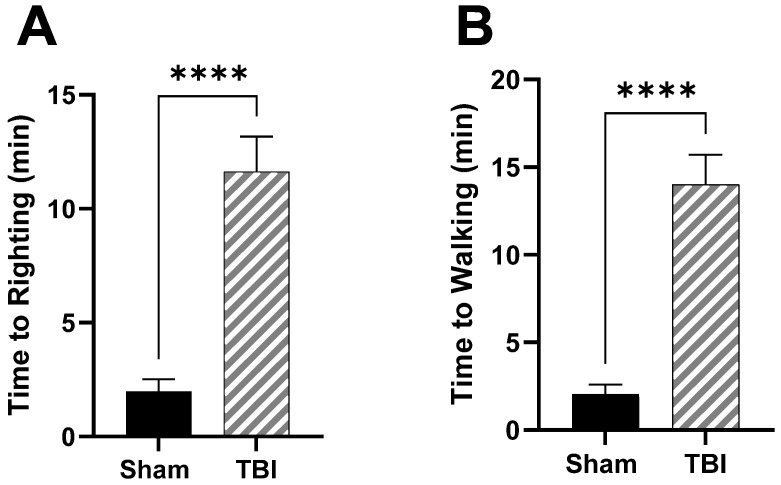
Righting and walking reflex post-injury. (**A**) Time to righting in min following TBI. (**B**) Time to walking in min following TBI. Data are presented as mean ± SEM. Student’s *t* test was used to determine statistical significance. Relevant statistical comparison reported by **** *p* < 0.001 (*n* = 6–8/group).

In the juvenile model of TBI, animals are either injured by the CHIMERA or sustain Sham procedures on PND 30. As previously reported, time to righting in TBI rats is significantly elevated, *p* < 0.0001 (Figure 1A). Time to walking is also elevated in TBI rats compared to Sham, *p* < 0.0001 (Figure 1B).

**Figure 2:**
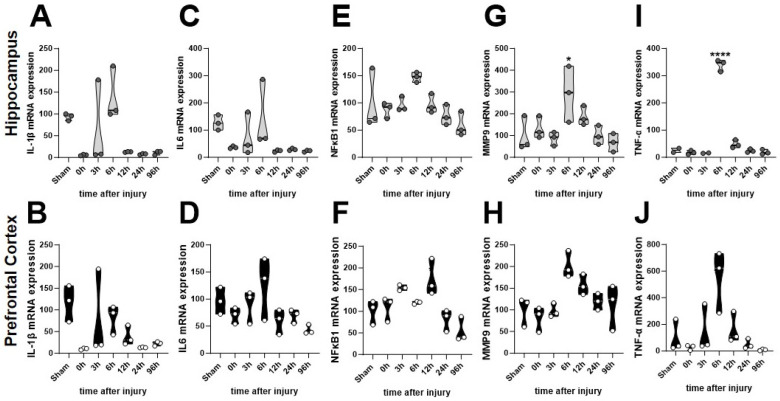
Time course of the mean gene expression of relevant cell markers impacted by TBI. Hippocampus is featured in black in the upper panels and prefrontal cortex in the bottom panels in gray. (**A**,**B**) IL1β (**C**,**D**) IL6 (**E**,**F**) NFκB1 (**G**,**H**) MMP9 (**I**,**J**) TNF-α. Statistical significance within each time point was determined through one-way analysis of variance with multiple comparisons followed by Tukey’s post hoc test. Relevant statistical comparison reported by * *p* < 0.05, **** *p* < 0.001.

To capture inflammatory marker changes in the hippocampus and prefrontal cortex, blocked regions of the brains were used for quantitative gene expression analysis. Despite peaks at 6 h, there were no significant differences in the time course of IL-1β in the hippocampus and prefrontal cortex, respectively (Figure 2A,B) or IL-6 (Figure 2C,D). Transcription factor NFκB1, an integrator of immune pathways, was significantly increased at 12 h in the prefrontal cortex compared to Sham, *p* < 0.05, but not the hippocampus (Figure 2E,F). Extracellular matrix protein MMP9 was elevated at 6 h in the hippocampus compared to Sham, *p* < 0.05, but only insignificantly peaked in the prefrontal cortex at 6 h (Figure 2G,H). TNF-α was robustly elevated in both the hippocampus, *p* < 0.0001, and the prefrontal cortex, *p* < 0.01, at 6 h post-injury compared to the Sham group (Figure 2I,J).

**Figure 3:**
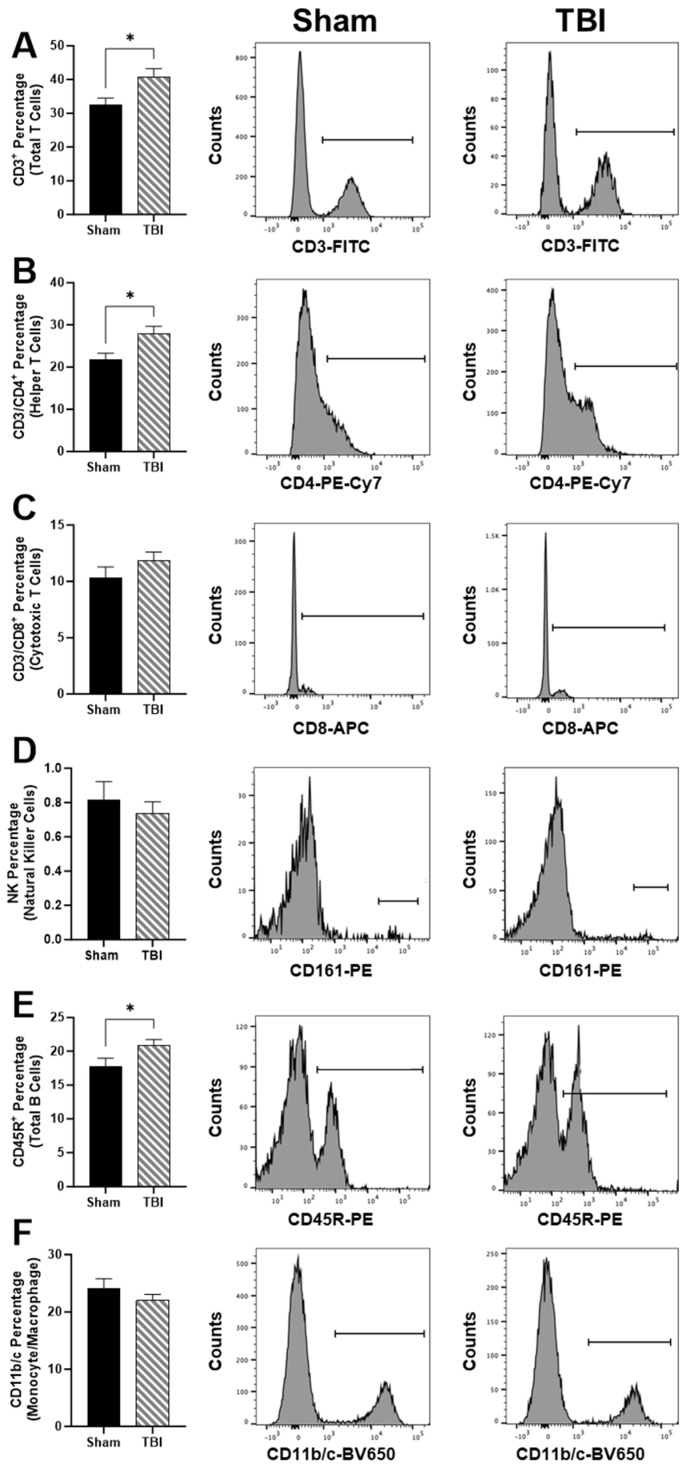
Peripheral blood lymphocytes at 48 h post-injury. Representative histograms and percentage of counts of (**A**) CD3^+^ Total T cells. (**B**) CD3/CD4^+^ helper T cells. (**C**) CD8/CD3^+^ cytotoxic T cells. (**D**) CD161^+^ natural killer cells. (**E**) CD45R^+^ Total B cells. (**F**) CD11b/c^+^ monocyte/macrophages. Data are presented as mean ± SEM. Student’s *t* test was used to determine statistical significance. Relevant statistical comparison reported by * *p* < 0.05. (*n* = 9–10/group).

We next investigated peripheral immune cell populations using cell sorting (Figure 3). TBI rats have elevated total T cells in circulation based on the CD3^+^ percentage, *p* < 0.05 (Figure 3A). In particular, CD3/CD4^+^ helper T cells were elevated in TBI compared to Sham, *p* < 0.05 (Figure 3). However, we did not measure any differences between CD3/CD8^+^ cytotoxic T cells (Figure 3C) or CD161^+^ natural killer (NK) cells percentage (Figure 3D). CD45R^+^ total B cell percentage was elevated in TBI compared to Sham, *p* < 0.05 (Figure 3E). No differences in CD11b/c monocyte/macrophage percentages were identified (Figure 3F).

**Figure 4:**
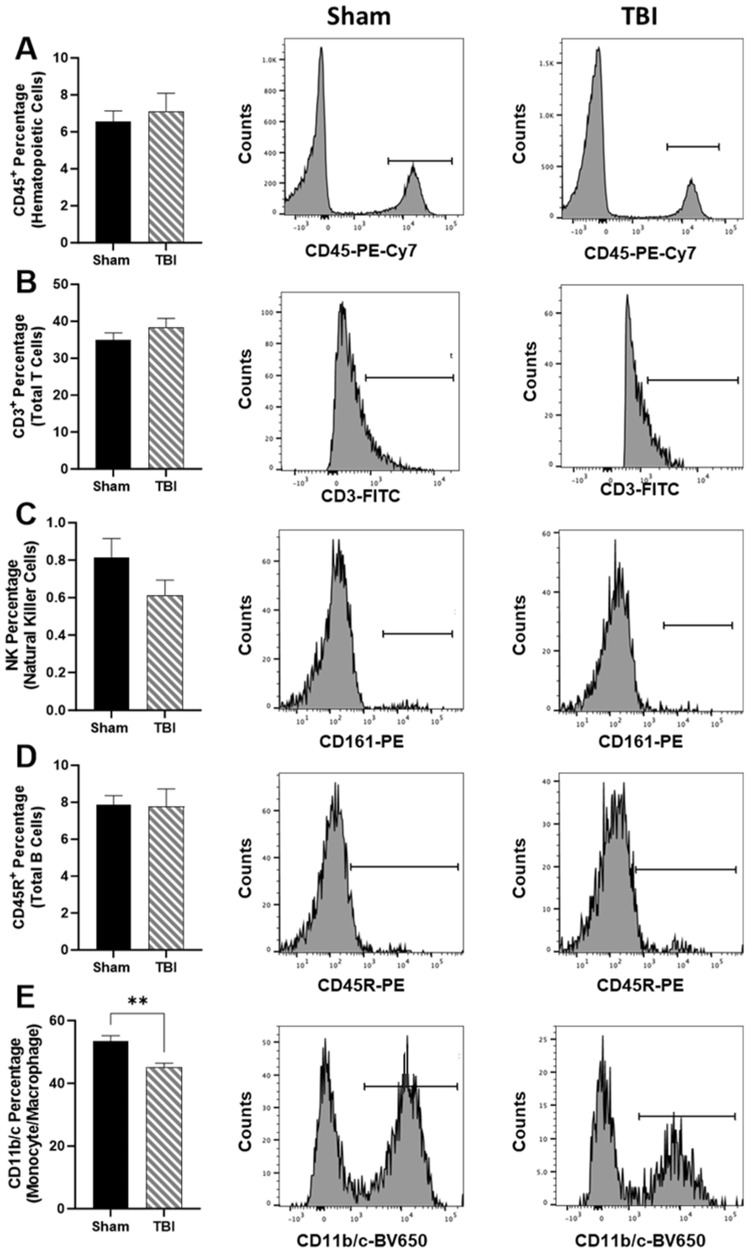
Cerebral hemisphere immune cell populations at 48 h post-injury. Representative histograms and percentage of counts of (**A**) CD45^+^ hematopoietic cells. (**B**) CD3^+^ Total T cells. (**C**) CD161^+^ natural killer cells. (**D**) CD45R^+^ Total B cells. (**E**) CD11b/c^+^ monocyte/macrophages. Data are presented as mean ± SEM. Student’s *t* test was used to determine statistical significance. Relevant statistical comparison reported by ** *p* < 0.01 (*n* = 9–10/group).

Using validated immune cell extraction protocols for the upper hemisphere of the brain, we did not measure differences in CD45^+^ hematopoietic cells (Figure 4A), CD3^+^ total T cells (Figure 4B), NK cells (Figure 4C), or CD45R^+^ total B cells (Figure 4D). Only CD11b/c^+^ monocyte/macrophage percentages were reduced in TBI in comparison to Sham, *p* < 0.01 (Figure 4E).

**Figure 5:**
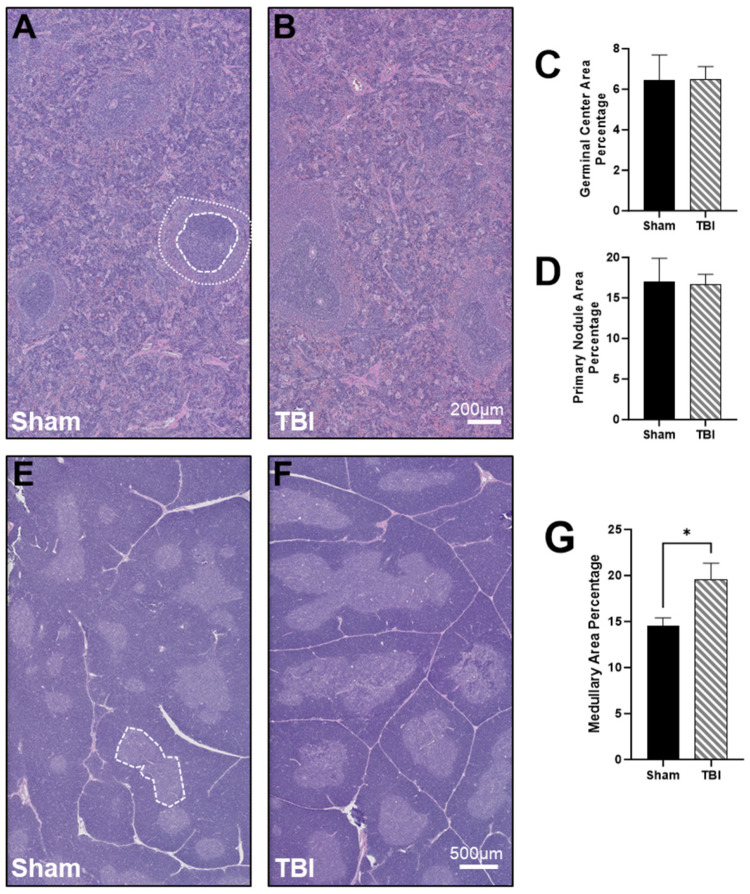
Splenic and thymic histology at 48 h post-injury. (**A**,**B**) Representative spleen H&E histology. The outer circle composed of smaller dashed lines indicates the primary nodule, while the inner circle composed of larger dashed lines indicates the germinal center. (**C**) A bar graph of percentage of splenic area composed of germinal centers. (**D**) A bar graph of percentage of splenic area composed of primary nodules. (**E**,**F**) Representative thymic H&E histology. The dashed line indicates the thymic medulla. (**G**) Bar graph of percentage of thymic area composed of thymic medulla. Data are presented as mean ± SEM. Student’s *t* test was used to determine statistical significance. Relevant statistical comparison reported by * *p* < 0.05. (*n* = 6–8/group).

Splenic tissue was extracted and processed for histologic quantification. Representative images of the spleen in Sham (Figure 5A) and TBI (Figure 5B) show a field of both germinal centers, primary nodules, and splenic cords. The germinal center area (Figure 5C) and primary nodule area (Figure 5D) were calculated as a percentage of the total splenic area, but neither was significantly different. Representative images of the thymus in Sham (Figure 5E) and TBI (Figure 5F) show both the thymic cortex and medulla. The medullary area was calculated as a percentage of the total thymic area, and the medullary area percentage was significantly greater in the TBI subjects, *p* < 0.05 (Figure 5G).

**Figure 6:**
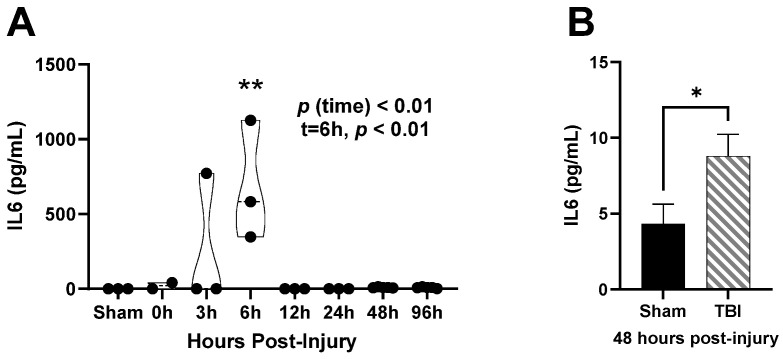
Plasma interleukin 6 (IL6) post-injury. (**A**) Time course of changes in plasma interleukin 6 levels. (**B**) Interleukin 6 levels 48 h post-injury. Data are presented as mean ± SEM. Statistical significance within each time point for time course data was determined through one-way analysis of variance with multiple comparisons followed by Tukey’s post hoc test. Student’s *t* test was used to determine statistical significance of 48 h post-injury data. Relevant statistical comparison reported by * *p* < 0.05, ** *p* < 0.01. (*n* = 6–8/group).

Using plasma from the time course studies, inflammatory cytokine IL-6 was measured in circulation (Figure 6A). There was a main effect of time, *p* (time) < 0.01, on IL-6 levels in the time course (Figure 6A). Sham levels were undetectable and continued to rise, peaking at 6 h post-injury, *p* < 0.01 (Figure 6A). Though not discernable in the graph, IL-6 levels remained elevated through 96 h (Figure 6A). Using the samples generated from the cell sorting study, we directly compared IL6 levels in circulation at 48 h post-injury (Figure 6B). IL-6 was significantly elevated in TBI subjects compared to Sham, *p* < 0.05 (Figure 6B).

## 4. Discussion

TBI is the result of a physical blow to the head; however, the damage produced is both architectural as well as inflammatory in nature. The objective of the current work was to identify the extent of immunological disruption to the juvenile brain and circulating immune system following CHIMERA-induced TBI in the acute phase. We show gene changes in the hippocampus (MMP9 and TNF-α) and prefrontal cortex (NFκB1 and TNF-α) and specifically reductions in CD11b/c^+^ monocytes/macrophages. The circulating total and helper T cell and total B cell percentages are elevated 2 days post-injury. Furthermore, thymic medullary expansion is occurring in TBI juvenile subjects.

In severe TBI, humans demonstrate increases in absolute number of leukocytes at 1- and 4-days post-injury in the peripheral blood [16]. Furthermore, both NK cells and cytotoxic T cells are substantially reduced at 4 days post-injury compared to healthy controls [16]. B cells remain stable throughout the 7 days of measurements [16]. Monocytes are elevated at 1-day post-injury compared to controls [16]. On the other hand, the present study did not find differences in NK, cytotoxic T cells, or monocytes at the time point sampled, while there were elevations in total B cells at 2 days post-injury.

To date, the literature is silent regarding the effect of CHIMERA-induced changes on immune cell populations, either centrally or peripherally. There are scattered reports of flow cytometry of immune cells in the juvenile population; these use a wide range of models and degrees of injury severity. Using a CCI model in PND 28 Sprague Dawley rats, peripheral monocytes and granulocytes were not altered compared to Sham at either 1- or 3-days post-injury [14]. Using an open-head weight drop model in adult C57Bl/6 mice, blood immune cells (B cells, T cells, NK cells, and neutrophils) remained unchanged during acute and chronic sampling periods [12]. Only monocytes were suppressed in the blood at 24 h post-injury and then during the remainder of the first-month post-injury [12]. Interestingly, while we found reductions in monocytes/macrophages in the brain, we did not identify a significant difference in this population in the blood. Again, in adult mice using a moderate injury in the CCI model, there was an increase acutely in monocytes and neutrophils, whereas lymphocytes were reduced at 1 and 3 days post-injury [13]. This is directly in contrast to our findings. Finally, in a repeated CCI injury model, adult Sprague Dawley rats initially show a reduction in T lymphocytes, with a rising level at 3, 7, and 14 days post-injury [11]. Unlike the previous examples in mice, which show opposite outcomes, the Sprague Dawley rats’ lymphocyte measurements more closely resemble our phenomenon.

Expansion in the total T-lymphocyte population in circulation, specifically helper T cells, that we identified may be supported by the significant thymic medulla area expansion we also report. The medulla of the thymus is responsible for T lymphocyte maturation and education, in particular in the juvenile, where involution of the thymus has not yet commenced. The thymus in juveniles has extraordinary flexibility to respond to various traumatic and inflammatory stimuli. The thymic reticular epithelial cells and dendritic cells provide guidance to the thymocytes during the maturation process through tissue-restricted antigens via MHCI and II by screening the cells for their T cell receptor specificity against self. The phenomenon of increased thymic medullary expansion following juvenile TBI has not been previously reported.

A far greater number of studies have investigated the changes in various parenchyma-residing immune cells following TBI. The microglia are self-renewing antigen-presenting cells in the central nervous system. In particular, they can present antigens to helper T cells in the brain. The resident microglia and the morphologic switch of the microglia between M1 and M2 phenotypes following injury are most commonly studied. In an adult mouse study using CCI, resident microglia are increased 7 days post-injury in the cortex [7]. Similarly, adult mouse CCI shows increases in microglia on the ipsilateral side of the brain at 2- and 14-days post-injury with parallel increases in PSD95^+^ immature microglia at 2 days, suggesting a de novo birth and maturation of resident microglia [17]. Using Marmarou’s weight drop model, Balb/c mice show macrophage increases at 1-, 2-, and 3-days post-injury, whereas microglia in the brain dramatically decrease [8]. Finally, using an open-head model of weight drop, monocytes in the brain are elevated at 24 h in severe, but not mild, TBI [10].

Our present work displays a reduction in monocytes/macrophages within the brain. Perivascular macrophages are significantly distinct from the microglia residing within the CNS. These perivascular macrophages exist at the interface between the brain and the blood and function to remove cellular debris and/or ingest cell components that are no longer needed. The reduction in the live monocytes/macrophage population residents in the brain 48 h after injury may be due to the clearing of these cells during architectural renovation following TBI.

The rat data are somewhat differing. In a male Sprague Dawley severe CCI model, microglia on the contralateral side are reduced as numbers of microglia on the ipsilateral side (same side as injury) are elevated at 24 h [18]. Using a closed-head weight drop model in female rats, single or repeat TBI shows no differences in resting or activated microglia or macrophages [9]. Though our study does not report changes to T and B lymphocytes in the cerebral cortex, we do report reductions in macrophages/monocytes, which is in contrast to the previously reported literature. The antibody used for the staining protocol should also stain neutrophils and potentially microglia. Many of these antibodies have been perfected in mouse models, where many of these studies are commonly performed.

Although we did not identify significant differences in IL6 mRNA expression in either the hippocampus or prefrontal cortex, there was a significant elevation in circulating IL6 levels at 6 h post-injury in comparison to Sham. Inflammatory cytokines increase after TBI have been commonly reported in both human and rodent models. In severe TBI, IL6, 8, and 10 are significantly elevated and together may be an early predictor of adverse outcomes in severe TBI patients [19]. Furthermore, polymorphisms in the promotor region of the *IL6* gene are associated with death or ICU stays in patients with severe TBI [20]. The multiplex of brain homogenates for inflammatory cytokines IL6, IL1β, and TNF-α show increases 6 h after injury using force thresholds similar to the ones we used with the CHIMERA [21], as well as in models of moderate to severe CHIMERA-induced TBI [22]. Although we did identify a significant increase in TNF-α mRNA expression at the same time point, we did not determine significant differences in IL6 or IL1β expression. Interestingly, TBI produced in IL-6 knockout mice results in poor behavior performance in open field tests and rotarod, suggesting overall beneficial actions of IL-6 increase to neurorehabilitation after TBI [23].

Though not addressed in the present work, the lymphatic system within the brain may be negatively affected by CHIMERA-induced TBI. The meningeal lymphatic vessels access the cerebrospinal fluid and produce drainage through a network of blind-ended capillaries that evacuate into larger vessels to remove waste, fluid, proteins, and interstitial fluid within the brain parenchyma. The glial endfeet, which are immunoreactively positive for water channel aquaporin-4, surround the lymphatic vasculature and contribute to the glymphatic drainage system of the brain. Both T and B lymphocytes, as well as antigen-presenting cells, travel through these lymphatic vessels [24]. The cervical lymph nodes may be crucial in supplying the brain with lymphocyte populations during various forms of trauma [24]. The model of TBI used in the current study is mild, and overt edema is not classically present in mild injuries. However, the contributions of the glymphatic system to immune responsiveness and facilitation of brain rehabilitation following injury deserve to be addressed.

## 5. Conclusions

The current study highlights the important changes to the immune cell populations in circulation and those immune cells homed to the cerebral cortices in the 48 hours following TBI. Our model does not feature any potential for sepsis or open-head. But in the human condition, the possibilities are endless for the additional injuries that may accompany the TBI. Thus, understanding the status of the immune system is important for overall recovery from injury.

## 6. Caveats and Future Directions

The impetus for the current studies rested on understanding immunological changes in the acute recovery phase following TBI with an eye for therapeutic targets during this recovery window. Thus, our investigation was limited in its scope. The Real-Time PCR time course experiments were used to give some indication of the timing for the cell sorting experiments. However, due to the small *n* size of the groups, there are limitations to these results. Furthermore, future investigations would utilize a variety of subacute and chronic time points to determine the long-lasting effect of TBI on both peripheral and central immune cells. Immunohistochemical detection of immune cells within the brain would assist in localizing the penumbra of the injury. Our previous studies highlighted the effect of obesogenic diets on various outcomes in pediatric rats. Given that these diets are chronically toxic to immune cells, we would anticipate that the changes in immune cells would be further exacerbated under high saturated fat conditions.

## Data Availability

The original data presented in the study are openly available at 10.6084/m9.figshare.25646400.

## Supplementary Materials

The following supporting information can be downloaded at: https://www.mdpi.com/article/10.3390/brainsci14040398/s1, Supplemental Figure S1: Flow-cytometry gating strategy. Representative images for the gating hierarchy in blood (A,B) and homogenized brain tissue. (A) Outline of the gating strategy to identify B cells, T cells and NK cells in peripheral blood. (B) Outline of the gating strategy for the cells of myeloid origin (C) Outline of the gating strategy for the immune cells obtained from the brain.
