# Supplementary material for: Cerebral and Peripheral Immune Cell Changes following Rodent Juvenile Traumatic Brain Injury
Source: Brain Sci. 2024 Apr 19;14(4):398. doi: 10.3390/brainsci14040398 (PMC11048136; doi:10.3390/brainsci14040398)
Supplement: Supplementary file 1 [file brainsci-14-00398-s001.zip › brainsci-2948380-supplementary.pptx]

## Slide 1
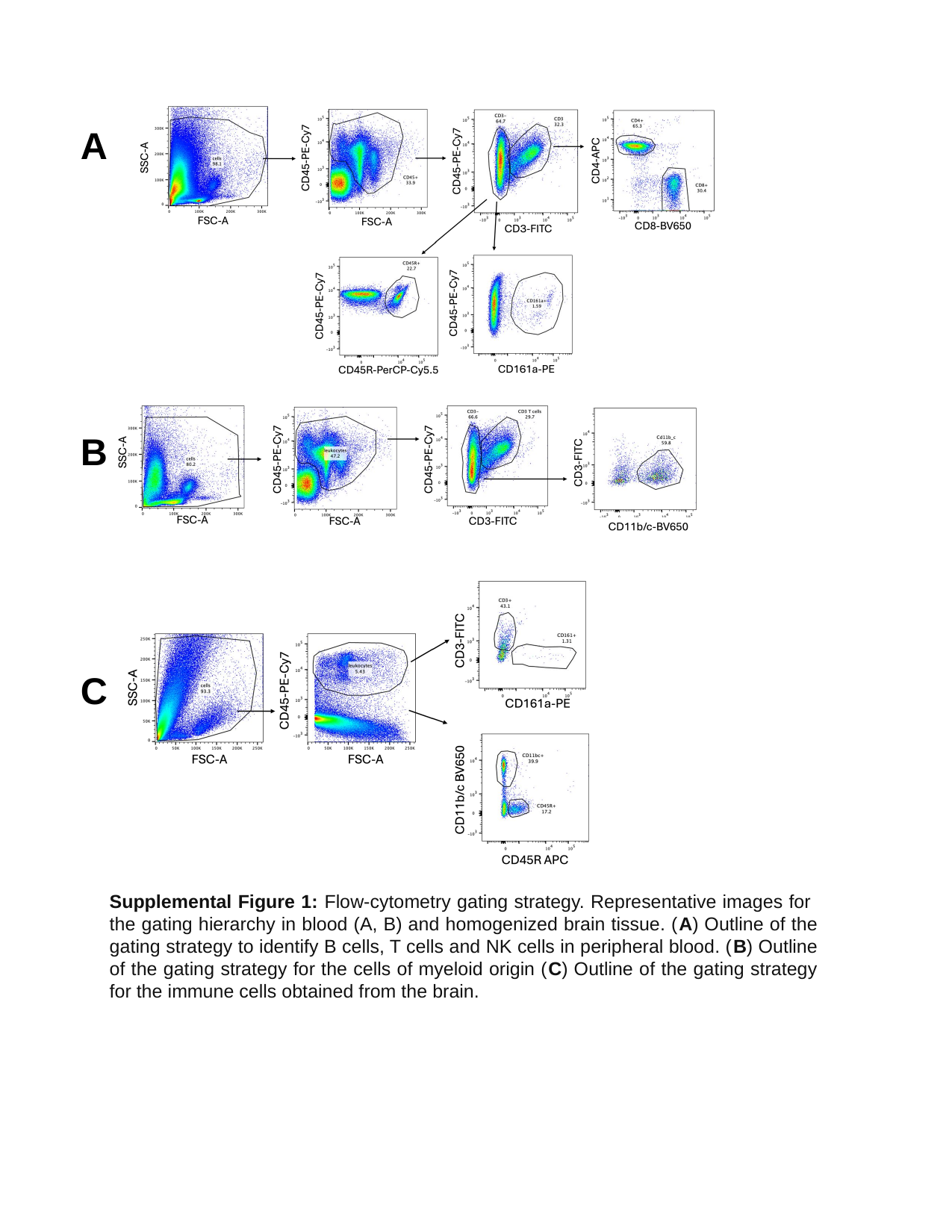

A
B
C
Supplemental Figure 1: Flow-cytometry gating strategy. Representative images for the gating hierarchy in blood (A, B) and homogenized brain tissue. (A) Outline of the gating strategy to identify B cells, T cells and NK cells in peripheral blood. (B) Outline of the gating strategy for the cells of myeloid origin (C) Outline of the gating strategy for the immune cells obtained from the brain.
